# Envisioning a high-quality health system in Nepal: if not now, when?

**DOI:** 10.1016/S2214-109X(18)30322-X

**Published:** 2018-09-05

**Authors:** Jigyasa Sharma, Amit Aryal, Gagan K Thapa

**Affiliations:** Department of Global Health and Population, Harvard TH Chan School of Public Health, Boston, MA 02115, USA (JS); Advisor to Member of Parliament Gagan K Thapa (AA) and Member of Parliament (GKT), Federal Parliament of Nepal, Kathmandu, Nepal

Nepal was one of the nine national Commissions that participated in *The Lancet Global Health* Commission on high-quality health systems in the Sustainable Development Goal (SDGs) era.^1^ which brought the widely acknowledged, yet insufficiently addressed, problem of systemic quality deficits in health systems to the forefront of the global health discourse.^1^ The HQSS Commission calls upon countries to make three interconnected commitments to reach the SDG goal of ‘health for all by 2025’. Countries are asked to first, invest in health systems that enhance health; second, to provide services valued by people; and third, to remain accountable for delivering high-quality care. The report of the Commission argues that adopting these commitments is primarily a political choice. In this Comment, we examine opportunities for action, the limits of the current health system, and the ways in which Nepal can meet the goals set out in the Commission.

Nepal’s commitment to universal health coverage (UHC) offers a starting point for rethinking the purpose and organisation of the health system and an opportunity to introduce the quality of care agenda into policy discourse. Expanding service coverage without quality will likely not result in improved health or financial risk protection—outcomes that are necessary to justify the substantial resource investment made towards achieving UHC. The agendas for universality and quality of care can and should be synergistic. The Commission recommends ensuring universal access to care with a minimum quality guarantee, and Nepal has already taken some steps in this direction: the 2017 National Health Insurance Act^2^ and the forthcoming National Health Institution Quality Authority Act^3^ provide the legal framework towards this goal.^4^

However, a promise for expanded coverage with a national quality guarantee is not a panacea for Nepal’s health system. To sustain this movement for a health system that is suited to meet shifting population health needs and preferences, disruption to the status quo with structural innovations is imperative. Nepal’s ongoing transition to federalism, coupled with its aspiration for UHC, opens a unique window of opportunity to feature the quality of the health system and its improvement as a priority in the national public policy agenda, and provides a new toolbox to embed quality in the core of health system policy and planning. Beyond agenda setting, this transition phase allows for macroscopic innovations at the central and federal level to realign, restructure, and redefine roles within and outside the health sector to realise policy reforms and institute a high-quality health system in Nepal.

The evolution of a health system determines how it functions and who it remains accountable to. The health system in Nepal has primarily developed against the backdrop of multiple donor-driven vertical programmes over the past few decades. Starting with the National Health Policy 1991, the importance of multisectoral coordination, decentralised planning and management, and overall health system strengthening has been repeatedly articulated, but the country’s aspiration for a robust health system has been unsatisfactorily realised.^5^ Consequently, the health system nowadays resembles a fragmented patchwork of various disease-centric vertical programmes of yesteryears, with inadequate focus on overall system strengthening. This is illustrated in the government’s budgetary practices (appendix): categories for spending are remnants of disease-centric vertical programmes, with scarce resources earmarked for addressing the rising burden of non-communicable diseases, mental health, and other emerging conditions.^6^

Although Nepal’s 2015 constitution guaranteed basic health care as a fundamental right, access to high-quality care remains a privilege.^7–9^ The poor quality of the health system can be gauged by one of the metrics suggested by the Commission—ie, use (or not) of the health system by heads of state. Nepali policymakers receiving medical care in neighbouring countries is now considered the norm. Additionally, people circumvent their nearest facility even for essential health services: about 70% of women in the Kaski district bypassed their nearest birth centres in favour of facilities with “adequate drugs and equipment” and “competent health staff”.^10^ These inadequacies reflect an obvious, but largely neglected breach in Nepal’s health system: it lacks people-centredness, which requires providing care that considers the preferences, needs, and aspirations of individuals and communities and that is accountable to users for improved health and value.^1,^^11^ What hinders progress towards people-centredness in Nepal’s health system is the continued prioritisation of disease-centred vertical programmes for defining health and health system functions.

The success of vertical programmes in curbing the scourge of select infectious diseases and the enormous advances in extending life expectancy, primarily through robust investments in improving child health, cannot be ignored. However, it is crucial to not let the success of vertical programmes warp our understanding of the capacity of the health system to consistently and sustainably provide care that enhances health and value for people. In tandem with global trends, the burden of poor health in Nepal has been compounded by the rising burden of non-communicable diseases, injuries, and chronic conditions. Acute, episodic care will not be sufficient for addressing these challenges; structural innovations that facilitate the seamless transfer of user information and responsibility within the health system to retain users and provide comprehensive longitudinal care are required. If the goal of the health system is to improve both health and people-centred outcomes, it will be important to move beyond programmatic proclivities of yesteryears and make judicious choices towards a strategy that drives necessary quality improvements and responsiveness into the health system.^12,^^13^

*Panel*: Towards a high-quality health system in Nepal: recommended next steps for the Ministry of Health

**Regulation**

- Enable regulatory structures to define and ensure minimum quality of care at both public and private health-care institutions, ranging from pharmacies to super-specialty hospitals
- Reform and regulate the procurement of medicines, medical equipment, and supplies by setting minimum quality criteria for import and use, and leverage economies of scale to reduce costs

**Public–private partnerships**

- Enhance public–private partnerships to fill gaps and address health system needs, from upgrading primary care capacity to developing centres of excellence at decentralised levels for research and specialty care
- Leverage the private sector to make progress towards universal health coverage by improving equity, efficiency, accountability, and quality of health-care services

**National Health Insurance Act**

- Reconcile supply-side and demand-side innovations across vertical programmes to bolster overall health system capacity and enhance access to high-quality care across health conditions and service platforms
- Institutionalise mechanisms to pay for quality rather than the narrower activity-based model under the National Health Insurance Act framework

**Education**

- Revamp preservice, in-service, and continuing medical education and training programmes by introducing a competency-based curriculum to promote evidence-based and respectful care delivery
- Invest in planning for a new cadre of public health-care providers, specialists, and researchers to address the shifting population health needs and aspirations

For decades, Nepal’s government and development partners—donors, non-governmental organisations, global health institutions—have committed to reducing the burden of poor health, especially among vulnerable and disadvantaged populations, with some degree of measurable success. However, a shortcoming of the existing model of predominantly disease-centric, donor driven, vertical programming is that, inadvertently but overwhelmingly, this model undermines the government’s capacity and responsibility towards ensuring that people have access to high-quality comprehensive health care. Instituting a high-quality health system will warrant bold leadership from the government and support from the development partners.

To this end, the government should shift gears to minimise its legacy function of service provision and enhance capacity for robust governance, with the purpose of guaranteeing equitable access to affordable and high-quality health care to its people. Capitalising on the opportunity that structures and functions of various tiers of government under federalism are being negotiated, the Ministry of Health should repurpose and reorient itself to push the envelope on governance and regulation by setting standards, producing guidelines, ensuring best practices, and strengthening the quality and cost-effectiveness of services offered by the health system (panel). Additionally, under the stewardship of the government, the benefits and limitations of the pyramidal system of health-care delivery can be assessed, and quality-focused service delivery redesign and mechanisms for enhancing accountability to people can be introduced.^1^

What role should the development partners assume? First, they should align their actions with the country’s vision and priorities for reforms towards a high-quality health system. Development partners should commit to a sustained engagement for realising these reforms. The need for vertical-horizontal synergy to strengthen the health system and upgrading health system foundations to deliver high-quality care should be a shared priority between the government and the donors, one that should be apparent in both agenda setting and resource allocation. This is crucial for ensuring the success of vertical programmes and addressing health system gaps without undermining the existing structures or reducing resources for system strengthening. Second, by introducing quality of care in tracking progress towards meeting global and national targets, including progress on UHC, development partners can elevate the importance and visibility of quality in the national policy sphere. Third, to institutionalise and raise the bar for accountability to citizens, development partners should invest in upgrading country capacity for transparent sharing of evidence on health system performance and responsiveness. Health system performance will be improved when standards set and enforced by the government are demanded by the people.^1^ Development partners can support these processes of generating demand for and supply of a high-quality health system.

High-quality health systems will lay the foundation for a healthier and more productive population. The current political juncture presents an opportune moment for Nepal to embark on the path to building a high-quality health system. Unwavering political commitment, structural innovations, and programmatic acceleration are essential for such policy reforms. To this end, the government must reverse the declining investment in health and progressively increase the share of annual budget towards health to pay for high-quality care. Systematic and sustained efforts from both government and development partners to embolden multisector planning, budgeting, implementation, monitoring, and evaluation are feasible and necessary. The people of Nepal are hopeful that the federal system of governance will better respond to their needs and expectations. In this historic moment, government and development partners must share a coherent policy vision and join efforts to redesign the health system with quality as its central organising principle.
